# Hypoglycemia in Oral Glucose Tolerance Test during Pregnancy and Risk for Type 2 Diabetes—A Five-Year Cohort Study

**DOI:** 10.3390/jcm13133806

**Published:** 2024-06-28

**Authors:** Noa Haggiag, Moran Rotman, Mordechai Hallak, Yoel Toledano, Rinat Gabbay-Benziv, Esther Maor-Sagie

**Affiliations:** 1 Department of Obstetrics and Gynecology, Hillel Yaffe Medical Center, Hadera 3820302, Israel; noale.hg@gmail.com (N.H.); moran.gawie7@gmail.com (M.R.); mottih@hymc.gov.il (M.H.); rinatg@hymc.gov.il (R.G.-B.); 2 The Ruth and Bruce Rappaport Faculty of Medicine, Technion—Israel Institute of Technology, Haifa 3200003, Israel; 3 Meuhedet HMO, Tel-Aviv, Israel; toledanoyoel@gmail.com

**Keywords:** diabetes mellitus, gestational diabetes, pregnancy, oral glucose tolerance test, reactive hypoglycemia

## Abstract

**Objective**: To evaluate the risk of progression to type 2 diabetes (T2D) following reactive hypoglycemia in 100 g oral glucose tolerance test (oGTT)**. Methods**: A retrospective analysis of parturients with up to 5-year follow-up postpartum. Data were extracted from the computerized laboratory system of Meuhedet, an Israeli HMO and cross-linked with the Israeli National Registry of Diabetes. Included were parturients with no prior diabetesand available oGTT values during pregnancy. Reactive hypoglycemia was defined as glucose levels lower than 60 mg/dL in at least one of 3 post-glucose load values in oGTT. The cohort was divided into 3 groups: normal glucose status, reactive hypoglycemia, and GDM. Maternal characteristics, laboratory data, and progression to T2D over 5 years were compared. Univariate and survival analyses assessed the adjusted hazard ratio for T2D, stratified by obesity **Results:** Among 14,122 parturients, 16.8% had reactive hypoglycemia, 71% had normal glucose status, and 12.2% had GDM. Adjusted for age, obesity, and hypertension, Parturients with reactive hypoglycemia had similar T2D risk compared to normal glucose status and a lower risk compared to GDM patients, regardless of obesity status. **Conclusions:** Reactive hypoglycemia during oGTT does not increase the risk of progressing to T2D.

## 1. Introduction

Type 2 diabetes (T2D) is a worldwide pandemic with increasing incidence globally. Early diagnosis is of great importance as individuals with undetected diabetes face an elevated risk of experiencing macrovascular and microvascular complications [1]. Gestational diabetes mellitus (GDM) is a significant risk factor for T2D. GDM is usually diagnosed by two step strategy including 50 g glucose challenge test (GCT) followed by 100 g oral glucose tolerance test (oGTT). Two abnormal values according to Coustan and Carpenter [2] are considered as GDM.

Reactive hypoglycemia during oGTT is a condition in which blood glucose levels drop to below-normal levels after glucose load. The exact definition of hypoglycemia is not uniform in the literature and varies according to the glucose values (45–60 mg/dL) and accompanying symptoms [3,4]. Accordingly, the prevalence of hypoglycemia during oGTT in pregnancy ranges between 3.6% and 11.3% in different reports [4,5,6,7].

The mechanism of reactive hypoglycemia during oGTT in pregnancy is not well understood and can be attributed to either dumping syndrome, gastrointestinal dysfunction, or idiopathic. Alternatively, hypoglycemia may result from hyperinsulinism that may precede T2D [8,9], as one of the earliest changes in T2D is first-phase insulin release loss, resulting in increased second-phase insulin release followed by reactive hypoglycemia.

Previous studies investigated the association between reactive hypoglycemia and pregnancy outcomes with conflicting results [4,5,6,7]. Reactive hypoglycemia is more prevalent in male fetuses, and younger and multiparous women [4,5]. Short term outcome of reactive hypoglycemia have been described, ranging between no adverse maternal or neonatal outcome to association with low APGAR scores, lower birth weight, small head circumference and short body length, and perinatal admission to the NICU [5,6]. This variability might indicate abnormal glucose metabolism during pregnancy. Assuming that is true, we expect that pregnant individuals with reactive hypoglycemia during oGTT will have higher progression rates to T2D years after pregnancy. However, data about the risk of developing future T2D after reactive hypoglycemia in oGTT during pregnancy is scarce. Therefore, in this study, we aimed to evaluate the association between reactive hypoglycemia in oGTT and the future development of T2D in up to 5-year follow-up as compared to pregnant women with normal glucose status or pregnanct women with GDM.

## 2. Materials and Methods

### 2.1. Study Design and Data Collection

A retrospective analysis aimed to evaluate the incidence of T2D among patients with a history of reactive hypoglycemia in oGTT performed during pregnancy. The study was approved by the local Institutional Review Board committee (10-18-08-21). Due to the retrospective nature of the study, informed consent was waived.

For this study, data were extracted from a dataset encompassing more than 5 years of laboratory data cross-tabulated with a pregnancy registry and integrated with the Israeli national diabetes registry (INDR) collected by Meuhedet Health Maintenance Organization (HMO). Meuhedet is one of four health insurance and medical services organizations that Israeli residents must belong to under Israel’s universal healthcare framework. The dataset included all individuals with documented pregnancy (by pregnancy registry) with last menstrual period (LMP) between 1 January 2017 and 31 December 2020. Maternal data included maternal age, body mass index (BMI), and diagnosis of hypertension, and delivery data included gestational age at delivery and neonatal sex. All clinical data were retrieved from the individual electronic medical records at the time of pregnancy. Laboratory data included first-trimester fasting glucose levels, 50-g glucose challenge test (GCT), and 100-g oGTT values. T2D diagnosis was retrieved from the INDR. As previously described [10], since 2012, all health medical organizations in Israel are requested by law to report cases of diabetes to the INDR. Data in this registry were linked to the pregnancy registry and the laboratory data of Mehuedet. Diabetes diagnosis is updated daily to the registry and defined as meeting one or more of the following criteria: (1) glycated hemoglobin greater than or equal to 6.5% (47.5 mmol/mol), (2) serum glucose concentrations greater than or equal to 200 mg/dL (11.1 mmol/L) in 2 tests performed at an interval of at least 1 month, and (3) 3 or more purchases of glucose-lowering medications. The registry has a sensitivity of 95% and the positive predictive value is 93% [10].

For this analysis, we included all pregnant individuals without a previous diagnosis of diabetes and available oGTT glucose values performed during pregnancy. We excluded all patients with fasting glucose levels >125 mg/dL and patients with missing values at oGTT. Only the first pregnancy was included for patients with more than one pregnancy during the study period to ensure the longest available follow-up time. Follow-up started at LMP and ended at the date of diabetes diagnosis, the date of data extraction (13 November 2022), or death—whichever came first.

By convention and according to Israeli guidelines, all parturients are recommended to undergo fasting glucose level in the first trimester to exclude overt diabetes (>125 mg/dL). Screening for GDM is recommended at 24–28 gestational weeks by the two-step approach: a 50-g screening glucose challenge test (GCT) followed by a diagnostic, 3-h, 100-g oGTT for the screen-positive patients (>140 mg/mL). Threshold values for GDM are consistent throughout pregnancy and defined according to the Carpenter & Coustan values [2] when GDM requires at least two out of four abnormal values.

Reactive hypoglycemia was defined as glucose levels lower than 60 mg/dL in at least one of 3 post-glucose load values in oGTT. The cohort was divided into 3 groups: normal glucose status, reactive hypoglycemia, and GDM. Maternal characteristics, laboratory data, and progression to T2D over 5 years were compared. Univariate and survival analyses assessed the adjusted hazard ratio for T2D, stratified by obesity.

### 2.2. Statistical Analysis

Maternal age and BMI were evaluated both as continuous variables and as categorical variables (with a cutoff of 35 and 40 years for age and 30 kg/m^2^ for BMI). Glucose levels, gestational age at delivery, and time to follow-up were treated as continuous variables while hypertension, GDM, neonatal gender, and T2DM were treated as categorical variables.

Categorical variables were compared using χ 2 tests and the Kruskal-Wallis test was used to test differences for continuous variables. All the tests were 2-tailed and *p* < 0.05 was considered statistically significant. Next, we applied survival analysis including Kaplan-Meier hazard curves and Cox proportional hazard models to estimate the hazard ratios (HRs) and 95% confidence intervals (CI) for incident T2D using the normal oGTT as the reference group.

## 3. Results

**Figure 1:**
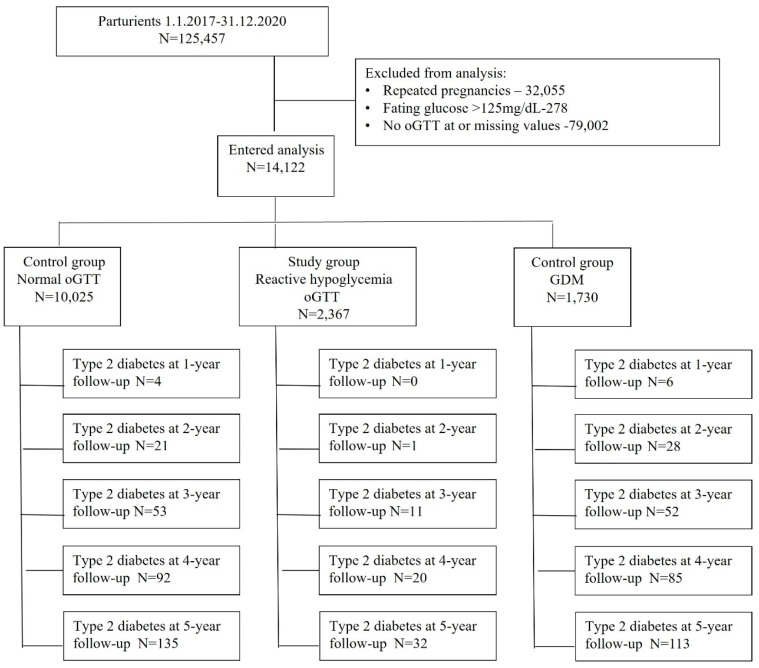
Study Cohort. oGTT—100-g oral glucose tolerance test; DM—diabetes mellitus; GDM—gestational diabetes.

Overall, our dataset included 14,122 pregnant individuals with available oGTT values and INDR data that entered analysis (Figure 1).

Stratified to study groups, 2367 (16.8%) patients had reactive hypoglycemia oGTT (study group), 11,025 (71%) patients had normal oGTT (1st control group) and 1730 (12.2%) had GDM (2nd control group). As shown in Table 1—among patients with reactive hypoglycemia, most had hypoglycemia at the 3-h blood evaluation (1 h—20/2367; 2 h 84/2367; and 3 h 2263/2367). Pregnant individuals with reactive hypoglycemia in oGTT were younger and had lower BMI compared to patients with GDM. Moreover, their glucose values throughout pregnancy including first-trimester fasting glucose, at GCT and all oGTT values were significantly lower compared to the GDM group (*p* < 0.001 for all). Compared to pregnant individuals with normal glucose status during pregnancy, patients with reactive hypoglycemia were older, leaner, and had lower glucose values (*p* < 0.001 for all).

As for risk for T2D, for the entire cohort, the median follow-up time was 4.24 years (IQR 3.25–5.04). Pregnant individuals with reactive hypoglycemia during oGTT had a statistically significant lower incidence of T2D separated by years and for the all study period compared to GDM. Moreover, there was no statistically significant difference between them and pregnant individuals with normal glucose status during pregnancy (Table 1).

Maternal age, BMI ≥ 30 kg/m^2^, and GDM were all statistically significant independent risk factors for T2D (*p* < 0.001 for all). Maternal age performed better when evaluated as a continuous variable and BMI performed better when evaluated as a categorical value, therefore were selected to enter the regression.

Next, we used survival analysis to account for the development of T2D over time and adjust the incidence of T2D to maternal age and BMI. After adjustment to confounders, pregnant individuals with reactive hypoglycemia had a similar risk for progression to T2D as compared to pregnant individuals with normal glucose status (HR 0.988 (95% CI 0.659–1.483), *p* > 0.05), Table 2.

**Figure 2:**
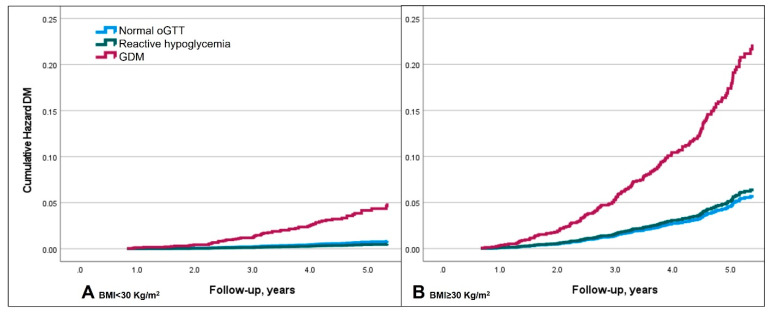
Reactive hypoglycemia in oGTT and cumulative risk for diabetes. Kaplan-Meier hazard curve. Reactive hypoglycemia and cumulative hazard risk for diabetes. (**A**)—for BMI < 30 kg/m^2^; (**B**)—for BMI ≥ 30 kg/m^2^. *p* value > 0.05 for reactive hypoglycemia in oGTT compared to normal oGTT (reference group. oGTT-100-g oral glucose tolerance test; DM—diabetes mellitus; GDM—gestational diabetes; BMI—body mass index.

Due to the significant association between BMI and T2D, which was also evident in our cohort, we further analyzed separately by obesity status. Reactive hypoglycemia at oGTT was not associated with risk for T2D both for patients with and without obesity as compared to patients with normal glucose tolerance (Figure 2).

## 4. Discussion

In this study, we aimed to evaluate the risk of T2D progression among pregnant individuals with reactive hypoglycemia during oGTT as compared to pregnant individuals with normal OGTT and GDM, in up to 5-year follow-up.

Our main findings were: (1) Prevalence of hypoglycemia in oGTT was 16.8% (2367/14,122). (2) Hypoglycemia was mainly noted 3 h following the sugar load. (3) Reactive hypoglycemia was associated with a lower risk of progression to T2D compared to patients with GDM in up to 5 years follow-up. (4) Regardless of obesity status, the risk of progressing to T2D was like that of pregnant individuals with normal OGTT.

The pathophysiology of reactive hypoglycemia remains contentious, with two opposing mechanisms proposed: reactive hyperinsulinemia versus tissue oversensitivity. Reactive hyperinsulinemia pertains to alterations in insulin secretion patterns observed in pregnant individuals with GDM [11]. Two distinct phases of insulin secretion are identified following oGTT. Among patients with GDM, the initial phase of insulin secretion is diminished while the second phase remains unaffected. Furthermore, in pregnant individuals with GDM, there is an exaggerated surge in plasma insulin and proinsulin secretion compared to non-GDM patients, a condition that doesn’t consistently revert to normal postpartum [11].

Since the loss of first-phase insulin release is one of the earliest changes in T2D development, prior studies [12] have suggested reactive hypoglycemia at oGTT as an early marker for undetected GDM. In T2D, the impairment of first-phase insulin secretion leads to postprandial hyperglycemia. Subsequently, there is a delayed and excessive secretion of second-phase insulin, resulting in later postprandial hypoglycemia due to prolonged elevated plasma insulin levels persisting after nutrient metabolism [13,14]. Furthermore, elevated insulin levels induce down-regulation of insulin post-receptor signals in muscles and fat, leading to decreased insulin sensitivity [15]. Other studies described impaired early insulin secretion as a marker of b cell dysfunction that appears prior to T2D diagnosis [16,17]. Therefore, delayed insulin secretion causing hypoglycemia during oGTT may indicate an abnormal hyper-insulinemic response. Interestingly, other research described an opposite phenomenon in which hypoglycemia post glucose load in non-pregnant patients was associated with increased insulin sensitivity and beta-cell dysfunction [18].

Prior studies point out that reactive hypoglycemia might increase insulin resistance, especially in young lean PCOS patients [9]. According to our study results, reactive hypoglycemia was not associated with an increased risk of progressing to T2D. Whether it is attributed to tissue oversensitivity is of no confidence. The discrimination between the two mechanisms could be carried out by real-time measurement of insulin and glucose levels to confirm the loss of first-phase insulin release.

Given that obesity stands as a significant risk factor for T2D and insulin resistance, it’s reasonable to anticipate that the subgroup analysis conducted based on maternal obesity status would at least partially distinguish between the groups. Finally, it should be considered that aside from these mechanisms, hypoglycemia in non-diabetic pregnancy can also be attributed to dumping syndrome, especially in pregnant individuals after bariatric surgeries [19], and does not necessarily indicate an inappropriate response to sugar load.

Our study has strengths and limitations. The study was based on a large cohort of pregnant individuals with high-quality data automatically generated from the laboratory cross-tabulated with INDR. Moreover, it benefits from a heterogenous population, with a distinction between individuals with and without obesity, and a validated statistic. Nevertheless, the study has several limitations mainly due to its retrospective nature. Most pregnant patients are younger than 40 years old and since we had a limited 5-year follow-up the overall risk of developing diabetes is relatively low. Missing data that could not be obtained, such as a family history of T2D or possible after-pregnancy interventions such as weight reduction /lifestyle modifications that might have changed the risk for T2D. Lastly, the INDR isn’t designed to identify patients with PCOS that might represent a unique group of reaction to oGTT.

In conclusion, according to our study results, reactive hypoglycemia during oGTT was not associated with an increased risk of T2D development in up to 5-year period after pregnancy. Pregnant individuals with reactive hypoglycemia during oGTT should be followed as non-diabetic patients.

## Figures and Tables

**Table 1:** Baseline demographic and progression to type 2 diabetes stratified by study groups.

	Control Group	Study Group	Control Group	
	Normal oGTT	Reactive Hypoglycemia oGTT	GDM *	*p* Value
	N = 11,025	N = 2367	N = 1730	
Maternal age, years	31.1 (26.7–35.6)	31.8 (27.3–36.1) ^$^	33.4 (28.7–37.9)	0.000
Age ≥ 35 years	2898 (28.9)	752 (31.8)	739 (42.7)	<0.001
Age ≥ 40 years	821 (8.2)	217 (9.2)	237 (13.7)	<0.001
BMI kg/m^2 #^	26.4 (23.1–30.7)	26.2 (23–30.7)	28.2 (24.5–32.5)	0.000
BMI ≥ 30	2702 (30.3)	614 (30.3)	645 (40.5)	<0.001
Hypertension	131 (1.3)	40 (1.7)	39 (2.3)	0.007
First-trimester fasting glucose, mg/dL	83 (77–87)	81 (77–87) ^$^	88 (81–94)	0.000
GCT, mg/dL ^#^	144 (123–155)	142 (110–154) ^$^	158 (146–173)	0.000
Fasting oGTT, mg/dL	77 (72–83)	76 (71–81) ^$^	87 (78–97)	0.000
1-h oGTT, mg/dL	143 (122–163)	135 (110–160) ^$^	197 (186–212)	0.000
2-h oGTT, mg/dL	116 (100–134)	100 (84–118) ^$^	170 (159–187)	0.000
3-h oGTT, mg/dL	92 (77–106)	50 (44–55) ^$^	114 (89–141)	0.000
oGTT week	28.1 (26–31.86)	27.7 (25.86–31.41) ^$^	27.4 (29.9–25.7)	0.000
Gestational age at delivery, weeks	39.7 (38.6–40.7)	39.9 (38.7–40.7) ^$^	39 (38–39.9)	0.000
Baby sex, male ^@^	5087 (54.2)	1247 (56.2)	817 (51)	0.007
Follow-up time ^&^	4.26 (3.47–5.09)	4.34 (3.47–5.09) ^$^	3.94 (2.9–4.9)	0.000
Type 2 DM, cumulative				
1-Year T2DM	4 (0)	0 (0)	6 (0.3)	<0.001
2- Year T2DM	21 (0.2)	1 (0)	28 (1.6)	<0.001
3-Year T2DM	53 (0.5)	11 (0.5)	52 (3.0)	<0.001
4-Year T2DM	92 (0.9)	20 (0.8)	85 (4.9)	<0.001
5-Year T2DM	135 (1.3)	32 (1.4)	113 (6.5)	<0.001

Data are presented as median (IQR) for continuous variables and n(%) for categorical values. Follow-up started at LMP and ended at the date of diabetes diagnosis, the date of data extraction (13 November 2022), or death—whichever came first. **^@^** gender results available for 13,207 deliveries; * GDM—defined as two abnormal values on oGTT (Carpenter & Coustan thresholds values [2]); ^#^ GCT available for 8565 patients; BMI—available for 12,719 ^&^ BMI—body mass index; oGTT-100-g oral glucose tolerance test; GDM—gestational diabetes; T2DM—type 2 diabetes mellitus; GCT—glucose challenge test. ^$^—Statistical significance relative to normal oGTT only.

**Table 2:** Cumulative incidence of diabetes during the study period.

	HR	95% CI	*p* Value
Maternal age, years	1.027	1.005–1.049	0.015
BMI ≥ 30	5.526	4.148–7.363	<0.001
oGTT—all normal values	***		<0.001
oGTT—reactive hypoglycemia	0.988	0.659–1.483	0.954
oGTT—GDM	4.232	3.229–5.547	<0.001

Cox regression analysis demonstrating the development of diabetes over the study period with maternal age and BMI as covariates. BMI—body mass index; OGTT-100-g oral glucose tolerance test; GDM—gestational diabetes; HR—hazard ratio; CI—confidence interval; ***—This group is the reference group and hence had no HR.

## Data Availability

The datasets employed and examined in this study can be obtained by contacting the corresponding author, through a reasonable request.
